# What Is the Main Cause of Shrinkage Porosity in AlSi7Mg0.6 Alloy Castings Obtained with an Increased Share of Secondary Materials?

**DOI:** 10.3390/ma19050910

**Published:** 2026-02-27

**Authors:** Jaroslaw Piatkowski, Katarzyna Nowinska, Tomasz Matula, Andrzej Nowrot

**Affiliations:** 1 Department of Material Technologies, Faculty of Materials Science, Silesian University of Technology, Krasinskiego 8, 40-019 Katowice, Poland; jaroslaw.piatkowski@polsl.pl; 2 Department of Applied Geology, Faculty of Mining, Safety Engineering and Industrial Automation, Silesian University of Technology, ul. Akademicka 2, 41-100 Gliwice, Poland; 3 Department of Metallurgy and Recycling, Faculty of Materials Science, Silesian University of Technology, Krasinskiego 8, 40-019 Katowice, Poland; tomasz.matula@polsl.pl; 4 Department of Electrical Engineering and Industrial Automation, Faculty of Mining, Safety Engineering and Industrial Automation, Silesian University of Technology, Akademicka 2, 44-100 Gliwice, Poland; andrzej.nowrot@polsl.pl

**Keywords:** Al-Si cast alloys, shrinkage porosity, iron phases, mechanical properties, microstructure of Al-Si alloys

## Abstract

Determining the causes of shrinkage porosity in Al-Si-Mg alloy castings with an increased proportion of secondary materials is very important and poses many problems. The reason for this is the existence of two opposing theories. One assumes that plate-like α-Al_5_FeSi (β-Fe) phase segregations cause shrinkage porosity. At the same time, the other believes that thin, double-layered oxide films with air-filled voids are responsible for the porosity. To address this question, the popular commercial alloy AlSi7Mg0.6 (EN AC-42200) was selected for testing. This alloy was cast into three series: with increasing content from 0.3 to 0.8 wt.% Fe and a constant content of approx. 0.1 wt.% Mn, the second with increasing iron and manganese contents (Mn/Fe = 1/2) (both series cast by gravity), and the third series under low pressure (approx. 0.15 MPa) with increasing content from 0.8 wt.% to 1.3 wt.% Fe and a constant content of approx. 0.1 wt.% Mn. Based on DTA (Derivative Thermal Analysis) and DSC (Differential Scanning Calorimetry) tests, the order of crystallizing components in various Mn/Fe combinations was determined. It has been found that the most unfavorable phases in gravity castings are the primary crystallizing β-Al_5_FeSi (β-Fe) phases (over 0.7 wt.% Fe), which are the leading cause of shrinkage porosity. After adding manganese to the alloy, thermal tests indicate that after the formation of α(Al) dendrites but before the eutectic α(Al) + β(Si), the Al_15_(Fe,Mn)_3_Si_2_ phase crystallizes. In die-cast samples, plate-like α-Al_5_FeSi (β-Fe) phase precipitates were also observed, but their share is small, and their average length does not exceed 20–30 µm. However, microstructural tests revealed the presence of rare oxides. It can therefore be assumed that in the AlSi7Mg0.6 alloy cast under pressure, the primary source of shrinkage porosity is not plate-like α-Al_5_FeSi (β-Fe) phase precipitates, but double-layer oxide films. In all cases, it was found that the Mg_2_Si phase formed at the end of crystallization does not affect shrinkage porosity.

## 1. Introduction

For several decades, there has been a growing demand for durable, lightweight Al-Si alloy castings for applications under variable thermal and mechanical conditions. This applies, among others, to the aviation and automotive industries, where lightweight castings are replacing heavy cast iron, cast steel, and steel products with favorable strength and specific stiffness [1]. The problem, however, is that during melting and casting processes, liquid aluminum alloys are susceptible to absorbing many metallic impurities, e.g., calcium, lithium, sodium, iron [2,3], and primarily gaseous impurities, the most important of which are oxides [4] and hydrogen [5,6].

This is because liquid aluminum has a high affinity for oxygen. When the surface of liquid aluminum comes into contact with atmospheric oxygen and moisture, oxidation occurs in milliseconds [7]. An additional factor contributing to the oxidation of liquid aluminum alloys is their transfer (e.g., from a furnace to a ladle or mold), as well as the high velocity of the liquid during casting. According to research [8], at a liquid flow velocity of more than 0.5 m·s^−1^, the flow becomes turbulent at the surface. This results in the formation of air pockets surrounded by oxide films, called “bifilms” by Campbell [9,10].

These films are essential because, during solidification, liquid aluminum reacts with the air it contains, forming additional aluminum oxides and nitrides [10]. The chemical reactions that occur during casting absorb the gas phase, while the advancing crystallization front can transport these bubbles into the liquid metal [11]. In addition, these thin oxide films formed during mold filling can become flattened or spherical due to four main factors, as reported in the studies [10]. Unfortunately, these are only theoretical considerations, as there is no direct experimental evidence to support them, especially since this requires 3-D X-ray microtomography with non-destructive scanning methods with micrometer resolution. Unfortunately, most studies on the role of oxide films in the formation of shrinkage porosity do not provide information on the iron content in the aluminum alloys tested, or the content is at the level of a few hundredths of a percent, as in alloy 2L99, or up to 0.2 wt.% in alloy A356 [7]. This raises the question: what role do oxide films play in the formation of shrinkage porosity in Al-Si alloys with an iron content of more than 0.2 wt.%? This applies in particular to alloys smelted from secondary materials, in which the iron content is even higher. In such alloys, do oxide impurities or crystallizing iron phases play a greater role in the formation of shrinkage porosity?

One of the few studies combining the presence of iron-rich phases with oxide inclusions has been published [12]. The authors chose the AlSi11Mg0.4 alloy with variable content ranging from 0.6 to 1.2 wt.% Fe and from 0.6 to 2.15 wt.% Mn for their research. Based on microstructure studies, they found that oxides such as MgO and Al_2_O_3_ in various allotropic forms (α, θ, δ, γ, κ) may constitute preferred substrates for the heterogeneous nucleation of α-Fe phases [12]. However, the authors do not mention the nucleation of β-Fe phases, in particular the porosity in the tested alloy, which may be important with respect to the identified cracks.

According to the authors of this study, these are legitimate questions, as iron is a common and highly harmful metallic impurity in Al-Si alloys, especially those cast from recycled materials. Its impact on mechanical properties, particularly plasticity, is particularly detrimental when it exceeds approximately 0.4 wt.% Fe (for gravity castings) [13,14] and 1.6 wt.% (for pressure castings) [15,16]. This is because iron has low solubility in the solid solution α(Al)—from 0.052% at 655 °C to 0.0052% at 450 °C [3]. For this reason, it has a strong tendency to combine with other elements, forming intermetallic phases with varying stoichiometry, structures, and sizes. As shown in studies, e.g., [17,18], the most unfavorable is β-Fe. Due to its low coherence with the matrix and its large size, it increases the alloy’s brittleness, hinders the machining of castings, and reduces tensile strength [19,20]. In addition, the plate-needle morphology of the β-Fe phase impairs the castability, ductility, and corrosion resistance of aluminum alloys [21].

The effect of iron on the porosity of Al-Si alloys was also the subject of research [22]. The analysis covered the AA309 alloy (AlSi5CuMg0.5) cast under low pressure in four combinations (with one and two sprues, with and without a cooling device), with 0.25 to 0.85 wt.% Fe. The results of the study [22] indicate that, despite the presence of gas impurities (oxides and hydrogen) in the alloy, iron is the primary cause of porosity, particularly under poor cooling and casting feed conditions. In addition, it was found that a content of more than 0.4 wt.% Fe causes a significant increase in porosity, regardless of the proportion of gas impurities in the alloys tested. Two iron-containing phases were identified in the microstructure of the tested alloy: stable β-Fe with a plate-like morphology and a transitional π-Al_8_Mg_3_FeSi_6_ phase with a dendritic structure [22]. Due to its dimensions and morphology, the β-Fe phase is responsible for increased porosity, as its plate-like precipitates limit the flow of liquid metal between the α(Al) dendrites, particularly along the second-order arms.

Similar conclusions were drawn in the study [21]. They show that among all alloying additives and gas impurities, iron-rich phases, mainly the β-Fe phase, are responsible for the formation of shrinkage porosity. To prevent this, elements that change the β-Fe phase into α-(Mn,Cr,Co) phases should be introduced into Al-Si alloys with increased iron content. The best described of these are namely α-Al_15_(Fe,Mn)_3_Si_2_ [16,23], α-Al_13_(Fe,Cr)_4_Si_4_ [24,25], and α-Al_15_(Fe,Co)_4_Si_2_ [26], or filtration techniques [27] can be used. Regardless of which method is more effective at eliminating the adverse effects of iron, a common feature is the unfavorable role of β-Fe phases in the formation of shrinkage porosity in Al-Si alloys with increased iron content. The authors of studies [17,18,19,20,21,22,23], demonstrating the harmful effects of β-Fe phases, do not specify the range of their crystallization, with most reporting a content of more than 0.4 wt.% Fe. This would be very helpful in explaining the role of β-Fe phases in the formation of porosity, especially since their length, depending on the iron content and heat dissipation conditions, can reach up to 500 µm [14,28].

It is therefore entirely reasonable to explain the formation of porosity in aluminum alloys, especially since it is the leading cause of reduced mechanical properties in both gravity [14,18] and pressure [16,25] castings made from them. More reservations are raised by determining the causes of shrinkage porosity in Al-Si alloys melted with an increased proportion of secondary materials. In such alloys, is porosity due to the presence of gas impurities (oxides and hydrogen) or the morphologically unfavorable β-Fe phases? How do casting conditions affect the formation of shrinkage porosity in secondary Al-Si alloys, which contain both increased levels of gaseous impurities and iron?

Attempting to answer these questions seems entirely logical. Therefore, it is justified to undertake further research to clarify the causes of shrinkage porosity in Al-Si alloy castings obtained with an increased proportion of secondary materials. The increase in the proportion of recycled scrap is not only due to the need to protect the environment by reducing energy consumption in production and waste, as well as limiting greenhouse gas emissions [29,30]. Due to declining supplies of primary raw materials and rising production costs, many aluminum alloy foundries are required to increase the proportion of recycled scrap, while maintaining current environmental standards.

The research aimed to identify the leading causes of shrinkage porosity in castings made of EN AC-42200 (AlSi7Mg0.6) alloy with increased iron content, resulting from an increased proportion of secondary materials (from 50 to 75%). An additional indirect objective is to identify which theory of porosity formation in Al-Si alloys with increased iron content is correct. Is it the one that assumes that shrinkage porosity is caused by the presence of plate-like β-Fe phase precipitates, or the one according to which the cause of porosity is thin, double-layered oxide films with empty air spaces inside them?

To achieve this goal, the scope of the research included as follows:

- Perform two series of smeltings with increasing content from 0.3 to 0.8 wt.% Fe and a constant content of approx. 0.1 wt.% Mn and increasing iron and manganese content (Mn/Fe = 1/2) cast by gravity;
- Perform one series of low-pressure smelting (approx. 0.15 MPa) with increasing content from 0.8 wt.% to 1.3 wt.% Fe and a constant content of approx. 0.1 wt.% Mn according to the adopted experiment plan;
- Thermal analysis DTA (Derivative Thermal Analysis) and DSC (Differential Scanning Calorimetry);
- Microstructure testing (including qualitative and quantitative analysis of shrinkage pores);

The novelties of this article include the following:

- Demonstrating the possible relationship between increased iron phase content—due to the higher use of charge scrap—and the development of shrinkage porosity in AlSi7Mg0.6 alloy castings. This applies to castings obtained by both gravity and low-pressure methods;
- Which theory of porosity formation in castings from secondary Al-Si alloys is correct? The one that assumes that shrinkage porosity results primarily from the presence of lamellar β-Fe phases, which hinder the flow of the liquid alloy during crystallization, or the one that assumes that porosity is caused solely by thin, bilayer oxide films?

Analysis of the presented considerations indicates that these theories are sometimes contradictory and do not fully explain the quantitative relationship between β-Fe phases and/or oxide films and porosity. Based on this, further research is warranted to elucidate the causes of shrinkage porosity in castings from Al-Si alloys with increased scrap content. This will expand our understanding of the potential influence of β-Fe phases on porosity, including the absence of these phases.

## 2. Materials and Methods

### 2.1. Research Materials and Methodology

#### 2.1.1. Methodology of Melting Alloys

**Figure 1:**
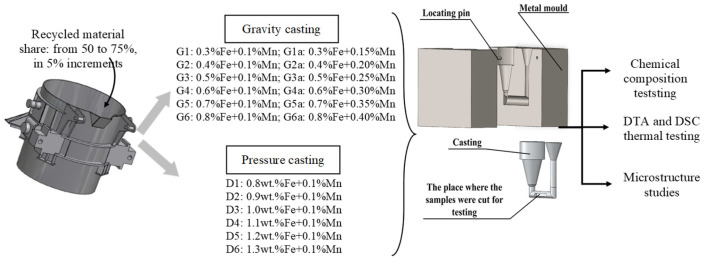
Schematic diagram of the experimental plan.

The literature review presented here shows that the main factor determining the causes of shrinkage porosity in aluminum alloys is the method of feeding liquid metal into the casting mold cavity. Therefore, a commercial AlSi7Mg0.6 alloy with increasing iron content was selected for testing. The alloy was cast under gravity and under pressure, and the proportions of β-Fe phases and shrinkage pores in both types of castings were compared. The increase in iron content was attributable to the increase in the proportion of secondary materials (from 50% to 75%, in 5% increments). The remaining charge consisted of primary alloys. Secondary materials mainly included the following: own circulating scrap, i.e., defective castings from pre-production tests, subjected to destructive testing (approx. 70%), and scrap purchased from other foundries and aluminum alloy processing plants (approx. 30%). The aluminum scrap was selected so that it differed mainly in iron content, while the other elements were within the tolerance limits of the AlSi7Mg0.6 alloy, i.e., 91.0–93.0% Al; 6.0–8.0% Si, 0.4–0.7% Mg; up to 0.2% Ti; up to 0.05% Cu (wt.%). During melting of secondary materials with primary ones, to prevent sedimentation and ensure the homogeneity of the metal bath, the alloy was stirred periodically, and the chemical composition was verified in separately cast samples. If necessary, missing elements were added. Once the alloy reached approximately 720 °C, it was modified with AlTi5B1 (0.05 wt.%) and refined with Rafglin-3 (Pedmo S.A., Tychy, Poland) at 0.2 wt.% of the charge weight. After checking the chemical composition for compliance with the assumed composition (Figure 1), six samples were cast:

- Gravitational casting into steel molds with increasing iron content from 0.3 wt.% Fe (G1) to 0.8 wt.% Fe (G6) and constant manganese content (approx. 0.1 wt.%);
- Gravity casting into a steel mold with increasing iron and manganese content (from G1a to G6a);
- Under low pressure from 0.8 wt.% Fe (D1) to 1.3 wt.% Fe (D6) on a Kurtz ND-GM B47 casting machine (Kurtz GmbH & Co. KG, Kreuzwertheim, Germany) with an average pouring speed of approx. 0.04 m·s^−1^.

Density was measured for all samples using the hydrostatic method. Samples for hydrogen content were taken from the melting furnace (for gravity castings) and from the preheating furnace of the die-casting machine (for LPC castings). Based on the sample mass measurements in air and in water, the density index was automatically determined to range from 2.4909 to 2.491 g·cm^3^.

Next, using the first gas bubble method on an Alu Compact II compact hydrogen tester (Seal, Warrington, UK), it was determined that the hydrogen content in all solid samples ranged from 0.22 to 0.28 cm^3^/100 g of alloy.

It was therefore assumed that since all samples had the same hydrogen content (and similar density), any influence on the development of shrinkage porosity could be attributed to the presence of oxide inclusions in the alloy being tested.

The castings were made at Superior Industries Production in Stalowa Wola, Poland. The experimental design and sampling locations are shown in Figure 1.

For gravity-fed castings, the crucible volume was approximately 2.5 L, enabling three castings to be obtained from a single melt (Figure 1). Therefore, three samples were obtained for each melt for chemical, thermal, mechanical, and structural testing. If the chemical composition results were consistent (within 0.05% of the standard deviation), the casting process was completed. The number and selection of low-pressure castings were the same.

Samples for chemical composition, DTA, DSC, and microstructure testing were cut from the location indicated in Figure 1 (round cross-section, Ø = 40 mm).

Chemical composition tests were performed on an optical emission spectrometer for quantitative elemental analysis, Foundry-Master Compact 01L00113 Worldwide Analytical System (Spectro Lab Analytical Instruments GmbH, Kleve, Germany). Ten measurements were taken; after discarding the two extreme values, the arithmetic mean was calculated from the remaining eight. The results were rounded to two decimal places.

#### 2.1.2. Methodology of DTA and DSC Methods

The DTA results were recorded using a standard QC 4080 thermal analysis probe (Heraeus Electro-Nite GmbH, Seekirchen, Austria) with a capacity of 110 cm^3^ and a heat removal rate (from T_liq._ to T_sol._) of approx. 40–50 °C·s^−1^. The freezing point (T) was recorded using a NiCr-NiAl thermocouple (type K) placed in the axis of the QC probe. DTA thermal derivative analysis was performed using a Crystaldigraph NT3-8K temperature recorder with MLab 2.1. software (Gliwice, Poland). The measuring set meets the requirements of EN61010 and EN60584 standards for temperature measurement. The curve of temperature change T over time τ (cooling curve T = f(τ)) and its first derivative dT/dτ = f’(τ) were recorded. Each time, the same volume of alloy, the same “smoothing” coefficients f’(τ), and similar melting, casting, and environmental conditions during measurement were used. The characteristic crystallization parameters were read from the curves: T_liq._ T_Emin._ (minimum crystallization temperature of eutectic α(Al) + β(Si)); T_E_ (crystallization temperature of eutectic α(Al) + β(Si)); T_EMg_ (eutectic-containing magnesium); T_Fe_ (crystallization temperature of phases containing iron); T_EFe,Mn_ (crystallization temperature of phases containing iron and manganese); T_sol._.

Phase transition studies using differential scanning calorimetry (DSC) were conducted on a Multi HTC high-temperature calorimeter (Setaram, Caluire-et-Cuire, France) in a 99.999% Ar (N 50) atmosphere in the temperature range from 450 to 700 °C. Cylindrical samples (weighing approx. 95 mg) were placed in an Al_2_O_3_ measuring crucible. The calorimeter was connected to the SetSoft 3.0. computer software (Setaram, France). The temperature was measured using a Pt-Rh10Pt thermocouple. High-purity Al_2_O_3_ powder was used as the reference substance. Before each measurement, the calorimeter was calibrated at a heating and cooling rate of 20 °C·min^−1^ (in accordance with ASTM E1269 for determining specific heat by differential scanning calorimetry). Repeated DSC tests showed that the temperature difference between heating and cooling was ±1 °C.

#### 2.1.3. Microstructure Research Methodology

Metallographic sections were prepared based on the recommendations of the Buehler expert system using Delta AbrasiMet Cutter-Buehler 102511 devices (Lake Bluff, IL, USA).

LM (Light Microscopy) microstructure tests were performed using an Olympus GX71MeF2 light microscope (Olympus Global EMC Ltd., Tipton, UK). SEM (Scanning Electron Microscope) tests were performed on a Hitachi S-3400N scanning electron microscope (Hitachi High-Technologies, Tokyo, Japan), equipped with an energy dispersive X-ray spectrometer (EDS, Thermo Fisher Scientific, Waltham, MA, USA), with Thermo Noran, Thermo Magna Ray wavelength dispersion, and INCAHKL Nordys backscattered electron diffraction (EBSD) detector (Thermo Fisher Scientific, Waltham, MA, USA). Ten images were taken, from which representative pictures of the microstructure of the AlSi7Mg0.6 alloy were selected. Some of the sections were etched in Fuss’s reagent.

A quantitative assessment of the microstructure was performed using quantitative metallography and image analysis in the AnalySis 12.2 software, with bright-field microscopy. For each sample, 10 images were taken in random fields of view. Binarization was performed using the Otsu method. The photos of the β-Fe phases were morphologically processed to map their size and shape accurately. The measurement was performed using the surface method, and the quantitative assessment of the morphology of β-Fe phase precipitates and shrinkage pores was conducted according to the procedure described in the studies [15].

The basis for distinguishing between pore types (contraction and gas) was adherence to the principles of quantitative metallography, the most important of which are as follows:

- Properly selected images of transformations of initial pore images into binary measurement;
- Methods for shadow correction (elimination) and image binarization before performing calculations;
- Estimating the necessary number of measurements required to obtain repeatable and reproducible test results;
- Assessment of inhomogeneity, i.e., the size, shape, and distribution of pores.

Pore inhomogeneity was assessed based on the following quantitative parameters:

- Fraction of the image area occupied by pores, given in %. Images with the same surface area were analyzed each time:
- Average pore surface area, μm^2^,
- Average pore circumference, μm.
- Dimensionless aspect ratio. The closer to unity, the more rounded the shape of an individual pore tends to be, which is a characteristic feature of gas pores. If this coefficient tends to zero, then the shape deviates from roundness, becoming dendritic, which is typical of contractile pores.

## 3. Results

### 3.1. Chemical Composition Test Results

The chemical composition results for the test samples are presented in Table 1.

### 3.2. DTA Test Research

**Figure 2:**
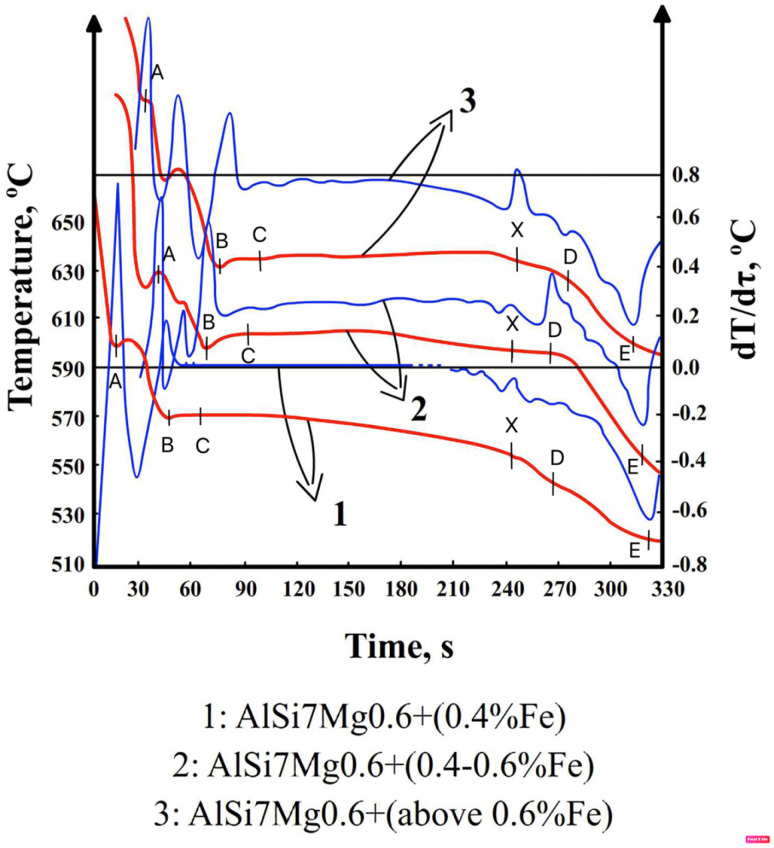
ATD curves of samples G2, G4, and G6 of the AlSi7Mg0.6 alloy.

**Figure 3:**
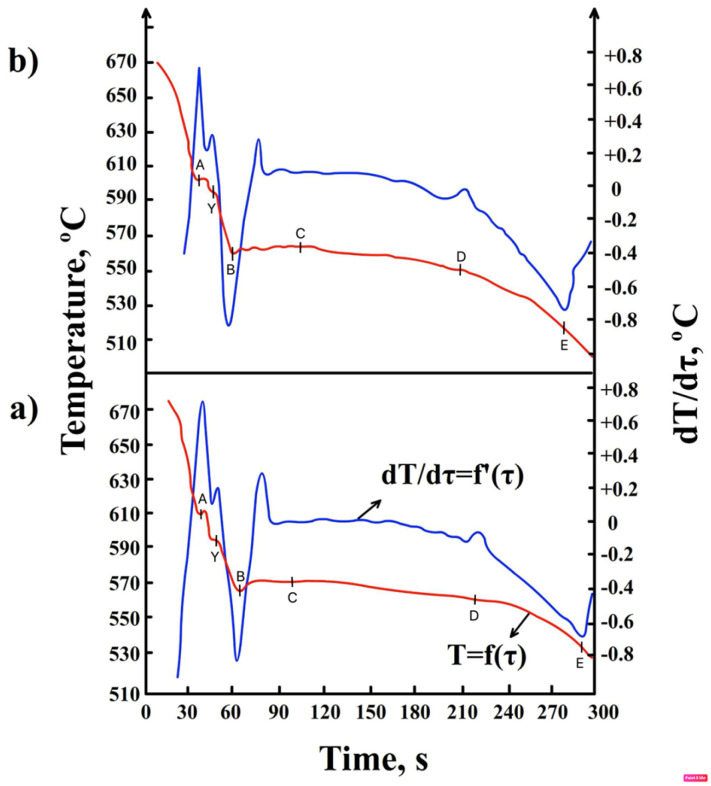
ATD curves of the AlSi7Mg0.6 alloy. (The explanation of points: A, B, C, D, E, and X and Y can be found in Table 2). (**a**) G2a sample (**b**) G4a sample.

The DTA graphs of samples G2, G4, and G6 are shown in Figure 2, while those of G2a and G4a are shown in Figure 3.

The characteristic crystallization temperatures of the gravity-cast AlSi7Mg0.6 alloy obtained from DTA tests are presented in Table 2.

The symbols in Figure 2 and Figure 3 and Table 2 represent the following:

– T = f(τ)—curve of temperature change T over time τ,—the so-called TA curve (Temperature Analysis);
– dT/dτ = f’(τ)—the first derivative of temperature change over time—the DTA (Derivative Thermal Analysis) curve;
– point A—the temperature at which the dendrites of a solid solution begin to crystallize α(Al)—T_liq._ = T_(α)_, °C;
– point B—minimum temperature (onset) of eutectic crystallization α(Al) + β(Si),—T_Emin(α+β)_, °C;
– point C—average eutectic crystallization temperature α(Al) + β(Si),—T_E(α+β)_,°C;
– point D—crystallization temperature of magnesium-rich intermetallic phases—T_E(Mg)_, °C;
– point E—end temperature of crystallization of the AlSi7Mg0.6 alloy (T_sol._), °C;
– point X—crystallization temperature of iron-rich intermetallic phases—T_Fe_, °C;
– point Y—crystallization temperature of intermetallic phases rich in iron and manganese—T_Fe,Mn_, °C.

### 3.3. DSC Test Research

**Figure 4:**
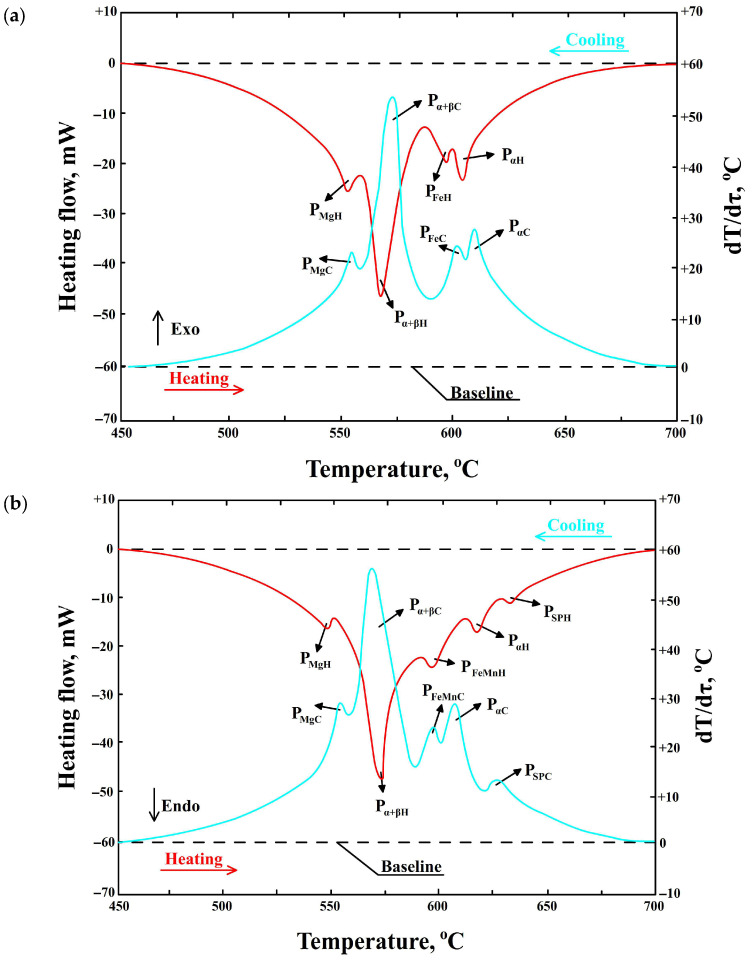
DSC curve of AlSi7Mg0.6 alloy during heating and cooling from the following: (**a**) alloy number G4 (0.61 wt.% Fe + 0.07 wt.% Mn); (**b**) G4a (0.58 wt.% Fe + 0.31 wt.% Mn).

To confirm the DTA results (or demonstrate any differences), DSC tests were performed. To limit the number of experiments, DSC curves were obtained for three samples with increasing iron content and constant manganese content (i.e., G2, G4, and G6) and for three samples with increasing iron and manganese content (i.e., G2a, G4a, and G6a). Examples of heating and cooling curves obtained by DSC for the AlSi7Mg0.6 alloy are shown in Figure 4.

A summary of the characteristic temperature ranges for the separation of structural components during heating and cooling, as determined by DSC, is presented in Table 3.

The symbols in Figure 4 represent the following:

– P_αH_—thermal effect resulting from the separation of dendrites of the a(Al) solid solution during heating, P_αC_—during cooling, mW;
– P_FeH_—thermal effect resulting from the precipitation of the Al_5_FeSi (β-Fe) phase during heating, P_FeC_—during cooling, mW;
– P_α+βH_—thermal effect resulting from the formation of double eutectic α(Al) + β(Si) during heating, P_α+βC_—during cooling, mW;
– P_MgH_—thermal effect resulting from the release of eutectic containing the Mg_2_Si phase during heating, P_MgC_—during cooling, mW;
– P_SPH_—thermal effect resulting from the release of sludge particles (SPs) during heating, P_SPC_—during cooling, mW;
– P_FeMnH_—thermal effect resulting from the precipitation of the α-Al_15_(Fe,Mn)_3_Si_2_ phase during heating, P_FeMnC_—during cooling, mW;
– Endo—endothermic reactions;
– Exo—exothermic reactions.

### 3.4. Microstructure Test Results

**Figure 5:**
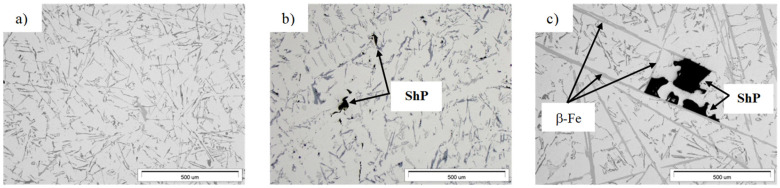
LM microstructure of the gravity-cast AlSi7Mg0.6 alloy: (**a**) sample G1 (0.29 wt.% Fe); (**b**) sample G3 (0.48 wt.% Fe); (**c**) G5 (0.69 wt.% Fe) (ShP—shrinkage porosity).

**Figure 6:**
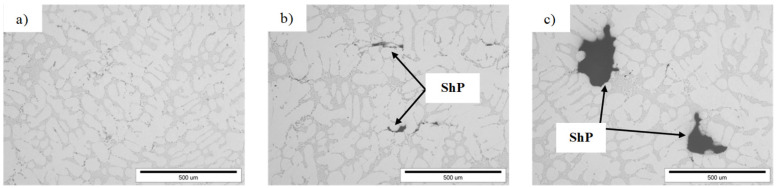
LM microstructure of the pressure-cast AlSi7Mg0.6 alloy: (**a**) sample D1 (0.81 wt.% Fe); (**b**) sample D3 (1.09 wt.% Fe); (**c**) D5 (1.21 wt.% Fe) (ShP—shrinkage porosity).

To identify the phases whose reactions were observed in the DTA and DSC curves, microstructure studies of the AlSi7Mg0.6 alloy cast by gravity (Figure 5) and under low pressure (Figure 6) were performed.

**Figure 7:**
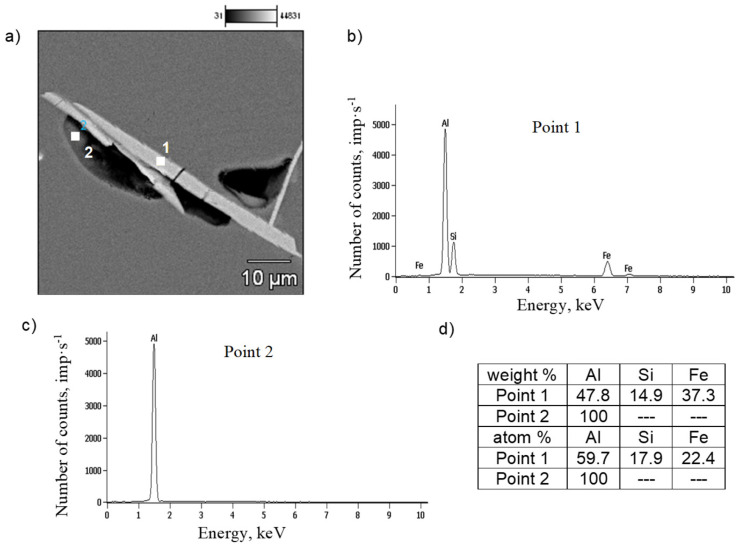
SEM microstructure (**a**) and chemical composition microanalysis results (**b**), (**c**) EDS spectra (**d**) at points 1 and 2 of gravity-cast AlSi7Mg0.6 alloy.

**Figure 8:**
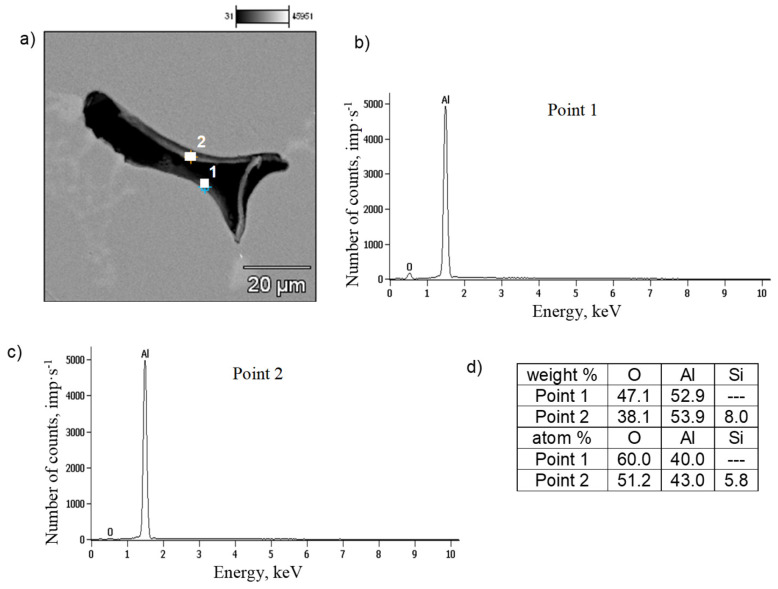
SEM microstructure (**a**) and chemical composition microanalysis results (**b**), (**c**) EDS spectra (**d**) at points 1 and 2 of the low-pressure cast AlSi7Mg0.6 alloy.

SEM tests were performed to identify the components of the AlSi7Mg0.6 alloy structure, and, in particular,, the potential material causes of shrinkage porosity. Several images of the microstructures of gravity-cast and low-pressure-cast samples were analyzed; representative examples are shown in Figure 7 and Figure 8.

**Figure 9:**
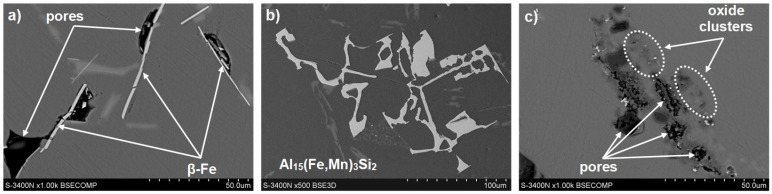
Representative microstructures of AlSi7Mg0.6 alloy cast: (**a**) gravity cast without manganese (sample no. G2); (**b**) gravity cast with manganese (sample no. G2a); (**c**) under low pressure (sample no. D2).

Analysis of the microstructure of the alloy under investigation revealed that in gravity-cast samples, needle-like precipitates of the β-Fe phase are located around shrinkage pores (Figure 9a). No such precipitates were found in the pressure-cast samples. Still, clusters of oxides were observed near shrinkage pores (Figure 9b).

**Figure 10:**
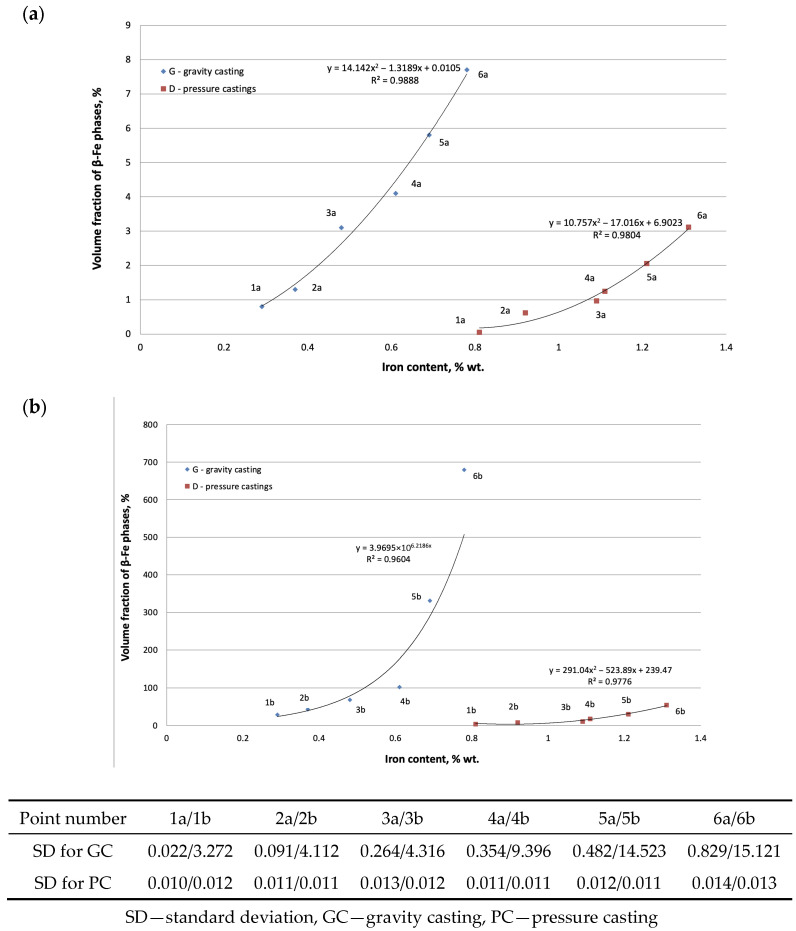
Change in (**a**) the average volume of β-Fe phase precipitates; (**b**) the length of β β-Fe phase precipitates as a function of iron content in the AlSi7Mg0.6 alloy cast by gravity and under low pressure.

**Figure 11:**
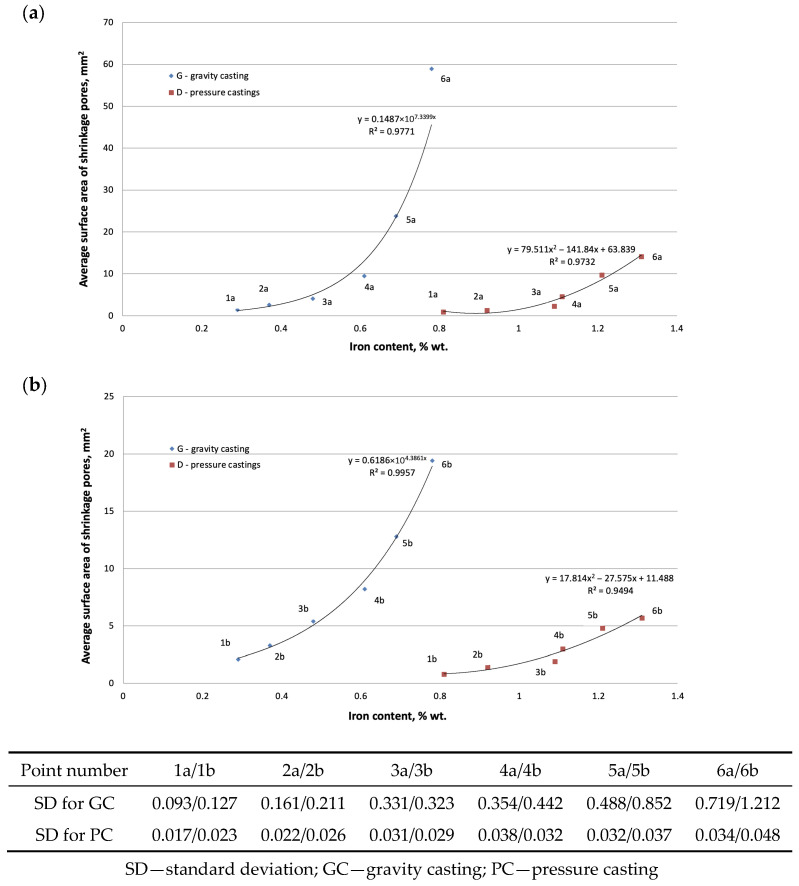
Change in (**a**) the average surface area of shrinkage pores; (**b**) the proportion of shrinkage pores as a function of iron content in the AlSi7Mg0.6 alloy cast by gravity and under low pressure.

The second part of the microstructure study of the AlSi7Mg0.6 alloy was a quantitative analysis of the β-Fe phase precipitates and shrinkage pores. The results of these studies are presented in Figure 10 and Figure 11.

## 4. Discussion

The research aimed to determine whether an increase in iron content resulting from a 50% to 75% increase in the proportion of secondary materials (circulating scrap) affects shrinkage porosity. The aim was therefore to answer the following question: which theory of porosity formation in Al-Si alloy castings is correct? Is it the one that indicates that shrinkage porosity results from the presence of plate-like (on the surface of needle-like castings) β-Fe phase precipitates in the alloy, which impede the flow of liquid metal, or the one according to which thin, double-layer oxide films are the cause of shrinkage porosity?

To try to answer this question, a popular commercial alloy, AlSi7Mg0.6, was selected for testing and cast in three batches (Figure 1):

1. Gravity with increasing content from 0.3 to 0.8 wt.% Fe and constant content of approx. 0.1 wt.% Mn—sample designation from G1 to G6;
2. Gravity with increasing iron content (from 0.3 to 0.8 wt.% Fe) and manganese (Mn/Fe = 1/2)—sample designation from G1a to G6a;
3. Under low pressure (approx. 0.15 MPa) with increasing content from 0.8 wt.% to 1.3 wt.% Fe and constant content of approx. 0.1 wt.% Mn—sample designation from D1 to D6.

The following tests were performed on the obtained samples:

- Thermal analysis using DTA and DSC methods—for gravity castings;
- Microstructure—for gravity and pressure castings.

To determine the crystallization sequence of structural components in AlSi7Mg0.6 alloy with increased scrap content, DTA (Derivative Thermal Analysis) and DSC (Differential Scanning Calorimetry) methods were selected to determine the order of crystallizing structural components of the AlSi7Mg0.6 alloy with an increased proportion of scrap. These are among the most widely used methods for thermal analysis of metal alloys. Their advantages (especially for DTA) are as follows:

- Measurement speed (up to several minutes);
- The possibility of testing under industrial conditions (on the production floor);
- Relatively simple interpretation of results;
- High versatility (determination of phase transformations and composition, metal purity, crystallization range, thermal stability, optimization of heat treatment parameters, structure prediction, etc.).

In addition, the ability to adjust the heating and cooling rates in the DSC method enables the identification of even phase transitions that absorb or release very small amounts of heat.

Thermal analysis using DTA and DSC methods shows that the crystallization of the AlSi7Mg0.6 alloy without manganese addition proceeds in the following order (Table 2 and Table 3):

- To a content of about 0.4 wt.% Fe:
  L → α(Al) → α(Al) + β(Si) → [α(Al + Al_5_FeSi + β(Si)]^EFe^ → [α(Al) + (Mg_2_Si) + β(Si)]^EMg^ → S (1)

- With a content of about 0.41 to 0.7 wt.% Fe:
  L → α(Al) → Al_5_FeSi → α(Al) + β(Si) → [α(Al) + (Mg_2_Si) + β(Si)]^EMg^ (2)

- With a content of more than 0.70 wt.% Fe:
  L → Al_5_FeSi → α(Al) → α(Al) + β(Si) → [α(Al) + (Mg_2_Si) + β(Si)]^E2^ (3)

If manganese (Mn/Fe = 1/2) is added to the AlSi7Mg0.6 alloy, the crystallization process (according to ATD) is as follows:

L → α(Al) → Al_15_(Fe,Mn)_3_Si_2_ → α(Al) + β(Si) → [α(Al) + (Mg_2_Si) + β(Si)]^EMg^ (4)

However, according to the DSC method,

L → SP → α(Al) → Al_15_(Fe,Mn)_3_Si_2_ → α(Al) + β(Si) → [α(Al) + Mg_2_Si + β(Si)]^EMg^ → S (5)

where

L—liquid state;

EFe—multicomponent eutectic containing the Al_5_FeSi phase;

EMg—multicomponent eutectic containing the Mg_2_Si phase;

SP—sludge particles;

S—solid state.

DTA studies show that in AlSi7Mg0.6 alloy without manganese (G1 to G6), up to approx. 0.4 wt.% Fe, β-Fe phase precipitates crystallize after or during the eutectic α(Al) + β(Si), which lasts until the crystallization of the eutectic containing the Mg_2_Si phase. The end of the Mg_2_Si phase crystallization is also the end of the crystallization of the entire AlSi7Mg0.6 alloy. The plate-needle β-Fe phases are short (up to 30 µm) and do not contribute to shrinkage porosity if the AlSi7Mg0.6 alloy contains between 0.41 and approximately 0.7 wt.% Fe, and the order of alloy crystallization changes: after the α(Al) dendrites but before the α(Al) + β(Si) eutectic, the β-Fe phase is formed. This is the so-called “pre-eutectic” crystallization of iron-rich phases. Their average length is approximately 30–200 µm. Suppose the iron content exceeds 0.71 wt.%, then the crystallization of β-Fe phase precipitates becomes primary (before α(Al) dendrites), and their average length exceeds 200 µm (most often from 300 to 700 µm) in gravity casting.

After adding manganese to the alloy (G1a to G6a), DTA tests indicate that after crystallization of α(Al) dendrites, a phase rich mainly in iron and manganese is formed (Figure 9b). Our own tests [30] show that this is the Al_15_(Fe,Mn)_3_Si_2_ phase, which crystallizes after α(Al) dendrites and before the α(Al) + β(Si) eutectic.

DSC studies also show that before the crystallization of α(Al) dendrites, sludge particles are formed (in the temperature range from 616 to 640 °C)—Table 3. Research [31] shows that these are “excess” particles rich mainly in iron and manganese, which have not been “bound” into the Al_15_(Fe,Mn)_3_Si_2_ phase. Their contribution to porosity formation is relatively insignificant. The crystallization temperature and morphology of sludge particles are described, among others, in publications [15,20] and our own research [31]. However, it should be noted that DTA did not detect their crystallization, which led to the conclusion that DSC studies are more accurate than DTA studies, especially for phases with a small amount of heat released.

The presented crystallization process of Al-Si-Mg alloys with increased iron content, both with and without manganese addition, is confirmed by the results of studies [21,22]. In addition, it was found that the Mg_2_Si phase formed at the end of crystallization does not affect shrinkage porosity.

Our own research [32] and a review of the literature [21,33] have shown that the main factor determining the causes of shrinkage porosity in aluminum alloys is the method of feeding liquid metal into the casting mold cavity. During gravity casting, unnecessary overheating and overflowing of metal should be avoided, and in the case of increased iron content, alloying agents should be used, e.g., containing manganese [16] or chromium [24], which changes the unfavorable morphology of the β-Fe phases to a dendritic or compact polygonal form [15]. This has also been confirmed by our own research [34]. In contrast, during pressure casting, casting parameters play an important role in reducing shrinkage porosity. As shown in studies [30], the most crucial factor is the pressure applied to the liquid metal during the first and second phases of the process. However, these publications did not specify the leading cause of shrinkage porosity. Microscopic studies of the AlSi7Mg0.6 alloy supplemented the DTA and DSC methods. The microstructure of the gravity-cast alloy under study shows that as the iron content increases, the quantitative proportion of the β-Fe phases increases, along with their length: from approx. 30 µm (up to 0.4 wt.% Fe) to approx. 700 µm (over 0.71 wt.% Fe) (Figure 5c and Figure 10). Such a significant increase in the length of the plate-like β-Fe phase precipitates is caused by the order of their crystallization relative to other components of the Al-Si-Mg alloy structure; as studies have shown, at a content of over 0.71 wt.% Fe, the β-Fe phases crystallize first, hence their length is the greatest. The initially formed (without any obstacles) “plates” of the β-Fe phases at the crystallization front thus hinder the flow of liquid metal to the interdendritic α(Al) areas, and the resulting empty spaces, after solidification, form shrinkage pores (Figure 5c and Figure 9a). It is also essential that no oxide inclusions were found in the microstructure of the tested gravity-cast alloy (regardless of iron content). On this basis, it can be concluded that in Al-Si-Mg alloys smelted with an increased proportion of secondary materials (and therefore with an increasing proportion of iron), the theory of shrinkage porosity formation as a result of the crystallization of plate-needle β-Fe phase precipitates is more likely. As already stated, these phases block the interdendritic melt flow channels, which is most evident at a content of more than 0.71 wt.% Fe. Their unfavorable morphology causes a deterioration in permeability at the crystallization front and, consequently, an increase in shrinkage porosity in castings made of Al-Si-Mg alloys from secondary materials.

The results obtained are consistent with studies [14,33], which found that as the iron content increases, the proportion of β-Fe phases increases, leading to an increase in shrinkage porosity clusters. The authors of studies [18,19] also state that this situation impairs the mechanical properties of castings, thereby underscoring the need to introduce additives such as manganese [16,21], chromium [24,25], or cobalt [26] into Al-Si alloys with increased iron content. Similar conclusions can be drawn from studies [27,33], which indicate that the porosity of gravity-cast Al-Si alloys is due to increased iron content.

A similar microstructure analysis was performed for the tested alloy cast under low pressure (approx. 0.15 MPa). The tests show that the increasing iron content (from 0.8 wt.% to 1.3 wt.%) does not cause a significant increase in the proportion of β-Fe phases, measured by their volume and length (Figure 10). However, the microstructures of the pressure-cast alloy indicate the presence of shrinkage pores (Figure 6b,c). However, no oxide films were detected in these microstructures, although areas with increased oxide density were quite rare. These are likely Al_2_O_3_-type oxides, which are formed as a result of aluminum oxidation. These are probably Al_2_O_3_-type oxides (Figure 8d), which are formed as a result of aluminum oxidation (aluminum comes into contact with oxygen or moisture contained in the air), hence their presence in micro-areas. However, it should be noted that the presence of reflections resulting from the oxygen content does not indicate the presence of oxide films in the alloy, but only the oxidation of the alloy surface. The Al_2_O_3_ layer remains thermodynamically stable on the surface of the liquid alloy. This is due to the lack of wettability between Al_2_O_3_ and the liquid aluminum alloy at 950 °C [5,7]. Studies [12,35,36] show that the average thickness of Al_2_O_3_ oxide layers is several dozen nanometers, which makes it very difficult to locate such films. Analysis of a large number of AlSi7Mg0.6 alloy microstructures has shown that this is possible, although very tedious and therefore difficult to prove. Taking into account the thermal expansion and volume shrinkage that accompany crystallization [7,10], tensile stresses arise in the oxide layers, causing surface cracks. This fact was used in the search for oxide films in the microstructures of the AlSi7Mg0.6 alloy. However, it should be noted that finding evidence for the existence of such oxide layers is very difficult. The difficulty lies in the fact that micro-computed tomography and a series of cross-sections, subjected to detailed analysis in micro-areas, would have to be used. Admittedly, plate-like β-Fe phase segregations were also found in the pressure-cast alloy under examination, but their share is small, and their average length does not exceed approximately 20–30 µm (Figure 10 and Figure 11). Based on microstructure studies, it can therefore be concluded that in the AlSi7Mg0.6 alloy cast under pressure, oxides are the primary source of shrinkage porosity. Observation of the melting and casting processes indicates that they most often occur during the transfer of the alloy from the furnace to the ladle or to the mold, as confirmed by studies [20,25]. As stated by Fox, Campbell, and Dispiran [35,36], among others, due to the high susceptibility of aluminum alloys to oxidation, it is essential to limit the transfer of the alloy and, in particular, the turbulence of the liquid metal in the first and second stages of pressure casting. Therefore, low piston speed (in the first stage) and low-pressure filling of the casting mold cavity (in the second stage) are recommended. This is confirmed by [30,37] among other studies. Thus, it can be concluded that in pressure-cast alloys, which are exposed to overflow and turbulence in the liquid state, the more likely theory regarding the causes of shrinkage porosity is the presence of oxide films in the alloy rather than morphologically unfavorable β-Fe phases. It should be emphasized, however, that the presented results are only indirect studies of the presence of oxide films as a cause of shrinkage pores in Al-Si-Mg alloy die castings. They confirm the accepted hypothesis about their influence on increasing shrinkage porosity in AlSi7Mg0.6 alloy die castings, but do not prove the mechanism of their action. However, explaining the role of oxide films as a cause of shrinkage pore formation requires a 3D characterization of the microstructure, including computed microtomography.

## 5. Conclusions

Research has shown that one of the most critical factors affecting shrinkage porosity in secondary aluminum alloys is the method of feeding the liquid alloy into the casting mold cavity and the cooling rate. In pressure casting, it is also essential to limit overflow of the alloy and, in particular, turbulence of the liquid metal during the first and second stages of the process. A low piston speed (in the first stage) and a moderate filling pressure in the casting mold cavity (in the second stage) are recommended.

Despite numerous studies on the causes of shrinkage porosity in Al-Si-Mg alloys with increased proportions of secondary materials (circulating scrap), there are many shortcomings in existing views on the subject.

Unfortunately, these views are often contradictory and do not explain many of the phenomena responsible for the formation of shrinkage porosity in Al-Si alloys, especially those with a high proportion of recycled scrap, which contains many impurities, the worst of which is iron. In gravity-cast Al-Si-Mg alloys with a content of up to approx. 0.4 wt.%, Fe and β-Fe phases do not pose a significant problem; hence, porosity is often accepted by customers. At a content of 0.41 to approximately 0.7 wt.% Fe, β-Fe phases (with an average length of 30 to 200 µm) form before the eutectic α(Al) + β(Si). In this case, single clusters of shrinkage porosity begin to appear. Still, they are not yet huge, as dendrites of the solid solution α(Al) have previously separated, effectively blocking (inhibiting) the growth of the β-Fe phases. At a content of more than 0.7 wt.% Fe, the β-Fe phases crystallize primarily, and hence their length is the greatest (from 300 to 700 µm), as they can grow freely and do not encounter any “obstacles.” In this case, the primary β-Fe phases block the free flow of the liquid alloy, leading to extensive clusters of shrinkage porosity with a morphology reflecting the later-crystallizing dendrites of the solid solution α(Al). It is then necessary to introduce additives based on elements that transform the unfavorable morphology of β-Fe precipitates into dendritic phases (with slow cooling) or compact polygons (with rapid cooling).

Regardless, microstructural studies of the AlSi7Mg0.6 alloy have shown that, in gravity castings, iron phases are responsible for shrinkage porosity.

In the case of pressure-cast Al-Si-Mg alloys, the presence of β-Fe phases is not as harmful, hence the permissible iron content is higher. The rapid crystallization of die castings into permanent molds means that the β-Fe phases are unable to reach a size large enough to impede the flow of liquid alloy at the crystallization front. However, the problem is the need to transfer the alloy from the furnace to the ladle, then to the die-casting machine’s pressing chamber. Inside the chamber, as a result of improperly selected process parameters (excessive piston speed and excessive pressure in the first and second stages of pouring), unnecessary turbulence of the liquid metal “front” may occur. As a result, the undesirable phenomenon of air occlusion [16,30] may occur, resulting in oxide films. As shown by microstructure studies of the AlSi7Mg0.6 alloy, these films occur around shrinkage pores. On this basis, it can be concluded that in the tested die-cast alloy, the primary source of shrinkage porosity is not plate-like β-Fe phase segregations, but double-layer oxides. Therefore, in the case of pressure-cast Al-Si alloys, the theory of porosity formation due to the presence of oxides in the alloy is more likely. In this case, alloying elements such as manganese and/or chromium are also introduced. Nonetheless, care must be taken not to exceed the permissible values of the so-called “slurry factor,” which is very unfavorable due to the presence of hard particles rich mainly in iron, manganese, and chromium [15,30,38].

Based on the research, the final conclusions are as follows:

1. Increased risk of crystallization: For Fe contents of 0.4 to 0.7 wt.%, the Al_5_FeSi phase (with a length of approximately 30 to 200 μm) precipitates along the Al(α) dendrites. Above the basic threshold (~0.7 wt.% Fe), this phase crystallizes primarily (reaching a length of 300 to 700 μm).
2. Precipitation of the Al_5_FeSi phase is inhibited by the properties of the interdendritic regions, which lead to increased shrinkage porosity. This can occur in a dispersed form; in gravity casting, the main cause of shrinkage porosity is Al_5_FeSi with a lamellar morphology.
3. The unfavorable use of the Al_5_FeSi phase can be introduced by adding manganese. The Al_5_FeSi cycle is in a smaller, compact Al_15_(Fe,Mn)_3_Si_2_ precipitate. A metallurgical solution.
4. During low-pressure feed transfer, the Al_5_FeSi phase is not as harmful, and the additional content available is higher. Rapid crystallization to the die forms results in a small phase (up to 30 µm) and is not hindered by the liquid alloy at the crystallization front.
5. Multi-stage liquid metal transport (furnace → ladle → injection chamber) introduces turbulence and aeration, using double-layer oxides (so-called “bifilms”). Therefore, during pressure transfer, after switching on the comparative feed, the Fe level is of secondary importance.
6. A Mn/Fe ratio of ≈1/2 should be used, which will lead to sludge effects. Their presence in Al-Si alloys melted with a separate scrap charge is unfavorable. This applies to the first pressure casting release.
7. Identification of oxide films in the microstructure of Al-Si-Mg alloys is very difficult and requires tedious micro-computed tomography studies and a series of cross-sections, subjected to detailed analysis in the micro-areas around the shrinkage pores.
8. The presented research results confirm the accepted hypothesis about the influence of oxide films on the development of shrinkage porosity in AlSi7Mg0.6 alloy pressure castings, but do not prove the mechanism of their influence. The detected presence of oxygen does not directly prove the morphology of oxide layers, but only the oxidation of the surface of Al-Si-Mg alloy castings. Therefore, we can only infer the presence of oxide layers in the microstructure of aluminum alloys, not directly observe them.

Next, when casting Al-Si-Mg alloys using scrap charge (secondary materials), the debate is resolved not by choosing a single theory, but by a specific method:

- For slow-action and quiescent processes (gravity casting), chemical composition control should be considered to preserve the morphology of the Al_5_FeSi phases. If necessary, manganese addition is required.
- If the method is used (pressure casting), a control process should be considered to prevent turbulence and oxide precipitation.

It should be noted that the presented results are preliminary studies aimed at identifying the main causes of shrinkage porosity. Due to the very small dimensions of the oxide films, their identification is extremely difficult. This requires specialized research equipment and 3D scanning methods on a manometric scale, followed by a tedious search for these films. Therefore, it can be concluded that the best method for identifying the causes of the formation and presence of shrinkage pores in the microstructure of pressure-treated Al-Si alloys is quantitative metallographic examination, supported by statistical correlation between the presence of oxides and the size and location of pores. Research in this area is currently underway.

In industrial conditions, where scale effects, longer furnace holding time, and the presence of other impurities in the scrap occur, the dimensions of the β-Fe phases may differ from those of samples cast under laboratory conditions. However, due to faster heat dissipation and the effect of pressure on the liquid alloy, they will certainly not be larger. This is confirmed by the test results shown in Figure 10 and Figure 11. The order of crystallization of the AlSi7Mg0.6 alloy components (relationships 1–5) observed in the tests will also remain unchanged, regardless of whether the conditions are laboratory or industrial.

It should also be noted that the occurrence of shrinkage porosity in Al-Si-Mg alloy castings with an increased proportion of secondary materials (circulating scrap) also requires analyses of the impact on hardness, mechanical, and technological properties (e.g., castability). However, this is the subject of further research, with results to be published soon.

## Figures and Tables

**Table 1:** Chemical composition of the AlSi7Mg0.6 cast alloy.

Sample No.	Element Content, wt.%									
	Si	Mg	Fe ^1^	Fe ^2^	Mn	Cu	Ni	Zn	Ti	Al
G1	6.93	0.57	0.3	0.29	0.08	0.05	0.04	≤0.08	0.12	rest
G2	7.11	0.62	0.4	0.37	0.06	0.07	0.03	≤0.08	0.15	rest
G3	7.08	0.60	0.5	0.48	0.06	0.03	0.04	≤0.09	0.13	rest
G4	6.96	0.59	0.6	0.61	0.07	0.03	0.02	≤0.10	0.13	rest
G5	6.97	0.61	0.7	0.69	0.08	0.05	0.02	≤0.06	0.15	rest
G6	7.08	0.61	0.8	0.78	0.09	0.04	0.02	≤0.10	0.18	rest
G1a	7.01	0.60	0.3	0.27	0.16	0.03	0.03	≤0.08	0.17	rest
G2a	6.96	0.59	0.4	0.40	0.21	0.05	0.04	≤0.09	0.15	rest
G3a	6.68	0.61	0.5	0.49	0.26	0.04	0.02	≤0.10	0.13	rest
G4a	7.02	0.60	0.6	0.58	0.31	0.03	0.03	≤0.10	0.16	rest
G5a	7.02	0.59	0.7	0.69	0.35	0.05	0.04	≤0.08	0.15	rest
G6a	6.96	0.61	0.8	0.81	0.41	0.04	0.02	≤0.09	0.13	rest
D1	7.05	0.62	0.8	0.81	0.40	0.04	0.04	≤0.10	0.15	rest
D2	7.12	0.60	0.9	0.92	0.45	0.03	0.03	≤0.06	0.18	rest
D3	7.20	0.59	1.0	1.09	0.49	0.04	0.02	≤0.10	0.15	rest
D4	7.18	0.62	1.1	1.11	0.51	0.03	0.01	≤0.08	0.13	rest
D5	6.98	0.61	1.2	1.21	0.60	0.05	0.03	≤0.09	0.16	rest
D6	7.02	0.59	1.3	1.31	0.65	0.04	0.01	≤0.10	0.16	rest

^1^—assumed iron content, ^2^—actual iron content in the alloy, G1–G6—marking of gravity-cast samples with increasing Fe content and constant Mn content, G1a–G6a—marking of gravity-cast samples with increasing Fe content and increasing Mn content, D1–D6—marking of samples cast under low pressure (approx. 0.15 MPa) with increasing Fe content and increasing Mn content.

**Table 2:** Characteristic crystallization temperatures of the AlSi7Mg0.6 alloy read from DTA diagrams.

Sample	Name of the Alloy and the Share of Iron and Manganese, wt.%	Points and Their Corresponding Crystallization Temperatures, °C						
		A T_liq._ = T(α)	B T_Emin(α+β)_	C T_E(α+β)_	D T_E(Mg)_	E T_sol._	X T_Fe_	Y T_Fe,Mn_
G1	AlSi7Mg0.6 + 0.3% Fe + 0.08% Mn	604	575	578	546	499	558	---
G2	AlSi7Mg0.6 + 0.4% Fe + 0.06% Mn	606	576	577	545	502	569	---
G3	AlSi7Mg0.6 + 0.5% Fe + 0.06% Mn	608	575	577	544	498	587	---
G4	AlSi7Mg0.6 + 0.6% Fe + 0.07% Mn	603	576	578	545	500	594	---
G5	AlSi7Mg0.6 + 0.7% Fe + 0.08% Mn	607	574	579	543	501	612	---
G6	AlSi7Mg0.6 + 0.8% Fe + 0.09% Mn	605	576	577	546	500	619	---
G1a	AlSi7Mg0.6 + 0.3% Fe + 0.16% Mn	607	576	578	543	499	---	581
G2a	AlSi7Mg0.6 + 0.4% Fe + 0.21% Mn	604	575	578	543	503	---	589
G3a	AlSi7Mg0.6 + 0.5% Fe + 0.26% Mn	608	576	579	544	502	---	592
G4a	AlSi7Mg0.6 + 0.6% Fe + 0.31% Mn	604	574	578	543	500	---	596
G5a	AlSi7Mg0.6 + 0.7% Fe + 0.35% Mn	606	575	577	546	502	---	599
G6a	AlSi7Mg0.6 + 0.8% Fe + 0.41% Mn	609	574	577	545	501	---	600

**Table 3:** DSC test results of the AlSi7Mg0.6 alloy during heating and cooling.

Sample	Heating							Cooling					
	T, °C	E(Mg)	α + β	FeMn	α(Al)	Fe	SP	SP	Fe	α(Al)	FeMn	α + β	E(Mg)
G2	T_start_	542	561	---	608	---	---	---	---	610	---	560	546
	T_end_	550	568	---	618	---	---	---	---	621	---	567	551
G4	T_start_	540	560	---	607	588	---	---	585	609	---	560	541
	T_end_	552	570	---	612	597	---	---	599	614	---	569	553
G6	T_start_	540	563	---	614	608	---	---	610	617	---	559	542
	T_end_	553	569	---	620	618	---	---	619	624	---	569	550
G2a	T_start_	540	562	573	608	---	---	---	---	610	575	568	543
	T_end_	551	570	582	614	---	---	---	---	618	580	571	549
G4a	T_start_	539	563	578	607	---	618	616	---	609	577	561	540
	T_end_	549	570	594	613	---	627	630	---	616	591	565	549
G6a	T_start_	541	560	581	609	---	623	620	---	610	582	560	542
	T_end_	548	569	603	616	---	640	639	---	615	601	565	550

T_start_—the temperature at which the chemical reaction (change) begins, °C; T_end_—end temperature of a chemical reaction (transformation), °C; SP—sludge particles.

## Data Availability

The original contributions presented in this study are included in the article. Further inquiries can be directed to the corresponding author.
